# GENETIC ARCHITECTURE OF ALZHEIMER'S DISEASE RISKS

**DOI:** 10.1093/geroni/igac059.003

**Published:** 2022-12-20

**Authors:** Abanish Singh, Konstantin Arbeev, Anatoliy Yashin, Igor Akushevich

**Affiliations:** Duke University School of Medicine, Durham, North Carolina, United States; Duke University, Durham, North Carolina, United States; Duke University, Durham, North Carolina, United States; Duke University, Durham, North Carolina, United States

## Abstract

More than 6 million people in the US live with Alzheimer's disease (AD) and related-dementia. There is a racial disparity in the prevalence of disease. However, its full genetic architecture and complex biological mechanisms are not yet understood. This study aimed to identify AD’s full genetic architecture. Hypothesizing that a large part of AD could be due to the complex interplay of multiple genes that may influence it directly or through comorbidity-risks, we performed a series of race-stratified GWASs on AD outcome and an array of correlated comorbidity-risks -- ADRD, depression, ischemic-stroke, cerebral-hypoperfusion, heart-failure, and cerebral-thromboembolism -- derived from Medicare records of HRS dataset’s White and Black samples. We prioritized AD GWAS signals based on their co-associations with comorbidity-risks. In White samples, we observed well-known genome-wide significant AD associations with SNPs in APOE, TOMM40 genes. In addition, we identified that 19 genic-region SNPs from the top 25 SNPs were mapped to 13 genes and that at least 5 genes were known for their association with AD or other neuropathic, neurological disorders. About one-third associations were replicated in Black samples. From the top 25 SNPs in Black samples, each of the 16 genic-region SNPs was mapped to a unique gene, which included 6 genes that were known for their association with other neurological, neuropathic, or psychiatric disorders. The genes resulted from the two races differed. These findings suggest that AD may result from a complex interplay of multiple genes, comorbidity-risks, and other brain-related disorders, which may differ for the two races.

